# Possibilities of Practical Use of Historical Distributions of Ash, Sulfur and Mercury Contents in Commercial Steam Coal of the USCB

**DOI:** 10.3390/e23070900

**Published:** 2021-07-15

**Authors:** Ireneusz Pyka, Wojciech Kempa, Krzysztof Wierzchowski

**Affiliations:** 1 Central Mining Institute, 40-166 Katowice, Poland; ipyka@gig.eu; 2 Department of Mathematics Applications and Methods for Artificial Intelligence, Faculty of Applied Mathematics, Silesian University of Technology, 44-100 Gliwice, Poland; wojciech.kempa@polsl.pl

**Keywords:** coal, coal parameters, probability distribution, variance, prediction

## Abstract

In the process of extracting hard coal, extensive databases are created on its quality parameters. A statistical assessment was made of the ash, sulfur, and mercury content of commercial coals produced in the Upper Silesian Coal Basin (USCB). The statistical methods applied: non-parametric tests of compatibility for two populations, parametric significance tests, and non-parametric tests of compatibility for the three populations, showed that the distributions of ash and sulfur content in 2014 and 2015 are comparable and the average values are similar. Statistical tests indicated significant differences in the mercury content distributions and their variances. This demonstrates the need for ongoing monitoring of mercury content in commercial coals, as a prediction of mercury content from historical data is hardly possible.

## 1. Introduction

The production of mineral raw materials involves the collection of different types of data on the quality characteristics of the raw materials. Data reporting allows the data to be used for statistical purposes, including forecasting and computer modeling. In the case of coal, quality characteristics are significant in many ways. The quality parameters: calorific value, sulfur content, ash content, moisture content, and volatile matter content, determine the price of coal either indicatively or directly [1,2]. Many quality parameters determine the behaviour of coal in the processes of its use and processing. Knowing them, as well as maintaining their values at the appropriate level, are essential for the proper conduct of these processes [3].

The qualitative characteristics of commercial coal, derived from the elemental composition of carbon matter and rocks surrounding the coal seams in the deposit, as well as the methods of coal mining and processing used are the basis for assessing the environmental impact of coal utilisation and processing. They determine the scale of the necessary preventive measures, for example, the emission of pollutants into the atmosphere [4]. Changes in coal quality parameters and the scale of required measures to reduce the environmental impact of coal utilisation and processing affect the costs of the processes. By knowing in advance about expected and unavoidable trends, changes in coal quality parameters, it is possible to plan preventive measures and optimise their costs.

Data on the basic price-forming parameters of coal, and those conditioning its behaviour during use, form extensive data sets. These figures include such parameters as ash, calorific value, and sulfur content. The mercury content of coal is an example of a qualitative characterisation that is performed much less frequently. More attention was paid to the mercury content of coal due to the reduction of mercury emissions to the environment [5,6]. European [7,8] and global efforts have led to the development and implementation of the Mercury Convention (Minamata Convention) [9].

The focus of this paper is the analysis of data describing three quality characteristics of hard steam coal, in two consecutive years. As a result of the project dedicated to the comprehensive evaluation of mercury content in Polish coal, a set of three quality characteristics was obtained for hard coal produced in the mines of USCB. These data are representative of the total production of hard steam coal, in terms of the structure of their production.

Selected quality parameter distributions were compared from year to year. The finding of homogeneity of probability distributions (or lack of homogeneity) can be used for practical estimates, for example of mean values of parameters and a measure of clustering around these values. This provides an estimate of the probability that the unit values of a given quality parameter will take values within the expected range. The results of such an analysis are of practical importance, especially for assessing and forecasting the technological and economic challenges arising from the variability of selected qualitative characteristics of the coal raw material.

The concept of entropy is often used in the study of the irregularity and degree of disorder of the statistical population. However, a more detailed study of the nature of a given phenomenon and the degree of its similarity with others requires the use of specific research tools, in particular properly selected statistical tests. The latter approach was used for this article.

## 2. Coal as an Energy Resource of Poland

**Figure 1:**
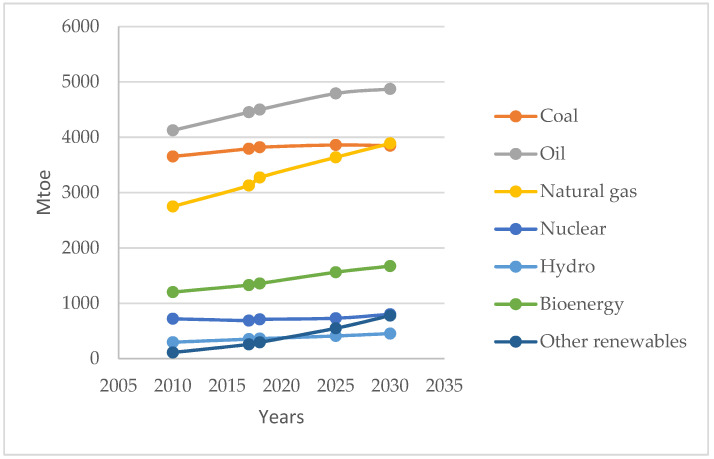
Energy demand by primary energy carriers-world.

Coal is one of the primary, non-renewable energy sources. Figure 1 shows the world demand for primary energy carriers from 2010 to 2018 (historical data) with a forecast until 2035.

This is the so-called “Stated Policies Scenario” forecast, which assumes the implementation of the current targets and plans for energy policies of various countries and regions of the world [10]. The apparent lower growth dynamics of coal consumption compared to other energy carriers, and even the forecasted minimal decrease in its consumption, do not significantly change the role of coal. It remains a key energy carrier for the global economy in the near term.

**Figure 2:**
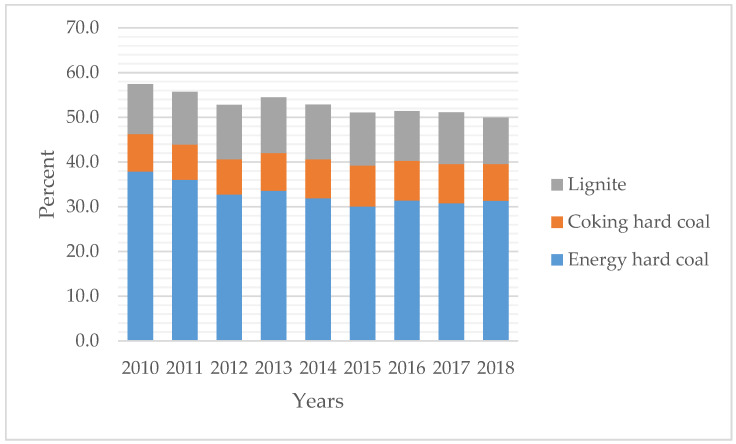
Share of coal in the consumption of primary energy carriers in Poland in 2010–2018.

In Poland, the role of coal in the economy is still crucial. Figure 2 shows the share of coal in the consumption of primary energy carriers in Poland for the years between 2010 and 2018.

As in the world, there is a decrease in the share of coal in this structure, but in 2018 the share was still around 50% [11]. About 31% of this is hard coal for energy purposes, about 8% hard coal for coking, and about 10% lignite [11]. In Poland, coal is used in many ways:

- in the energy generation sector (power plants, combined heat and power plants, heat plants, and district heating boilers of the commercial power industry)—about 60% of total consumption; including hard coal and almost all lignite mined in Poland,
- in industry and construction, including coking plants—about 24% of consumption; hard coal only,
- in households—about 13% of consumption; hard coal only.

About 83% of the domestic hard coal production comes from USCB. The results of the analysis are influenced by several factors, resulting from the centuries-long tradition of coal mining in USCB. Values and variability of the data given to the analysis are not solely the result of variability in the quality characteristics of the coal in the deposit. In addition, the following can be mentioned:

- carrying out production in different parts of partially depleted deposits, frequently moving with mining to another part of the deposit, as well as reaching deeper, geologically older parts of the deposit,
- varying degrees of raw coal enrichment in individual mines, resulting in a change in the quality characteristics of commercial products,
- the complex structure of mines’ final products as a result of multiple uses of hard coal in Poland,
- closure of mines with depleted deposits or with too high extracting costs.

## 3. Research Material

Data describing three quality characteristics of hard steam coal for energy purposes produced in the mines of USCB in two consecutive years were analysed.

The data were obtained as a result of determinations made on samples of coal representative of each commercial size grades produced in the mines of USCB. In cases of smalls, when commercial products were created by mixing several components, samples of these components were taken. Each time, the total sample consists of several dozen portions. Most often, portions of coal from each size grade or component were taken over two weeks, on each working shift. The same sampling process was repeated in the following two years. In each case, total samples were taken from all the commercial assortments produced in that year.

The collected samples were subjected to a reduction process and samples for testing were separated from them following the provisions of PN-ISO 13909-4:2005—Preparation of test samples. Quality parameters were determined for each sample following the standards:

- solid fuels: determination of ash by gravimetric method PN-ISO 1171,
- total sulfur content PN-G-04584:2001,
- the certified internal procedure, elaborated in Główny Instytut Górnictwa No. SC-1.PB.23 applying the Cold-Vapor Atomic Absorption Spectrometry, using the analyzer MA-2000 of Nippon Instrument Corporation. It is a fully automated measurement system for the determination of mercury content in solid materials, gases, and liquids through sample combustion or evaporation.

As a result of the activities described above, two groups of data were collected, for ash, total sulfur, and mercury content, with a count of 174 elements, each with a grouping variable denoting the consecutive year of production.

## 4. Statistical Research Methodology

Statistical analysis of the distributions of ash, sulfur, and mercury contents of commercial thermal coal in USCB was carried out using the STATISTICA PL software package for statistical calculations and analyses (version 13.3). In particular, the following statistical analysis tools were used in the study:

- non-parametric tests of compatibility for two populations: the Wald–Wolfowitz series test, the Kolmogorov–Smirnov test, and the Mann–Whitney U test,
- parametric significance tests for two means (*t*-test) and two variances (F-test, Levene’s test, and Brown–Forsythe test),
- non-parametric tests of compatibility for the three populations: the Kruskal–Wallis test and the median test.

A typical significance level of 0.05 was assumed for all statistical tests. The choice of these statistical tools is related to the purpose of the analysis [12,13,14,15,16]. Since the hypotheses related to the homogeneity (compatibility) of the distributions of the selected characteristics will be verified, the most important tests for the consistency of the distributions in the case of two and three populations (items 1 and 3) were applied. In addition, significance tests for two means and two variances were used to compare the two most important numerical characteristics of the distributions (mean and variance) (Section 2).

## 5. Comparative Analysis of Ash, Sulfur, and Mercury Content Distributions in Successive Years

The analysis aimed to confirm the representativeness of the data from each year (see Supplementary Materials), thus assessing the compatibility of the distributions of ash, sulfur, and mercury content from one year to the next. The two-dimensional random sample for each of the three study variables (ash, sulfur, and mercury content) had 174 elements.

The following non-parametric tests were used in the study: the Wald–Wolfowitz series test, the Kolmogorov–Smirnov test, and the Mann–Whitney U test. The results are presented in Table 1, Table 2 and Table 3.

None of the three tests conducted gave grounds to reject the hypothesis that the distributions of ash content in 2014 and 2015 were compatible. This is probably since ash is constantly monitored in the production of commercial coals. Ash is one of the most important qualities and pricing parameters of coal. We reject the null hypothesis that the distributions of sulfur content are compatible only for the Wald–Wolfowitz series test. In the case of the other tests, there are no grounds for rejecting them. In all the tests performed, we reject the null hypothesis that the distributions of mercury content in each year are compatible. This demonstrates the high variability of mercury distribution in the coals produced.

## 6. Parametric Tests for Equality of Means and Variances in Successive Years

Parametric tests were additionally carried out, verifying the null hypothesis of equality of the means and variances of the contents of each of the parameters studied in 2014 and 2015. The *t*-test was used for verification of equality of means, and the following tests were used for variance: the F-test, the Levene’s test (marked as L), and the Brown–Forsythe test (marked as B–F) for two variances. The *t*-test was used in the version with separate variance estimation (no assumption of compatibility of variances). The results are grouped in Table 4.

In the case of the distributions of ash and sulfur content, there are no grounds to reject the hypotheses of equality of means and variances in successive years. The distributions of mercury content in 2014 and 2015 are significantly different as to the mean value, the conclusion is therefore similar to the non-parametric tests used earlier. For the distribution of mercury content, there are insufficient grounds to reject the null hypothesis of equality of variance.

## 7. Compatibility Tests for the Distributions of all Parameters Tested

The following tests were used in the analysis: the Kruskal–Wallis rank test, and the median test (each time to analyse the compatibility of the three distributions: ash, sulfur, and mercury content). The analysis was carried out separately for 2014 and 2015. Table 5 and Table 6 contain the test results for the data from 2014. For the 2015 data, the test results are identical (*p*-values are 0.000).

The results of both tests cause the rejection of the null hypothesis of compatibility (sameness) of the distributions of all three parameters.

## 8. Conclusions

The results of the tests carried out show that the distributions of ash and sulfur contents of commercial steam coals in successive years are comparable. In the case of ash, none of the tests causes the rejection of the hypothesis of their compatibility, both in 2014 and 2015. In the case of sulfur content, only one of the tests rejects the null hypothesis that the distributions are compatible, while the others indicate that they are compatible. This conclusion is confirmed by parametric tests for two means and two variances. The average values of ash are close to each other and are 12.9% and 14.2%, respectively, in two consecutive years. The average value of total sulfur in both years is the same at 0.65%. The variance values of ash and sulfur in both cases are also similar. This means that, with a probability of 95%, the mean values of the two quality parameters can be assumed to be equal.

Statistical tests indicate significant differences in mercury distributions. The average mercury content was 97.5 µg/kg in 2014 and 78.8 µg/kg in 2015. This indicates a large variability in the average level of mercury content in commercial coals in both years and confirms the results of earlier work [17], where variability in mercury content in selected coal seams of USCB was shown based on studies of documentary seam samples. This indirectly leads to large uncertainties in the estimation of atmospheric mercury emissions from the electricity generation sector [18].

The analysis of historical data allows for a good prediction of coal quality concerning the ash and sulfur content of commercial coal from USCB. In the case of mercury, ongoing monitoring is advisable. Prediction of mercury content in coal, based on historical data, is virtually impossible.

## Figures and Tables

**Table 1:** Results of the Wald–Wolfowitz series test of the compatibility of distributions in 2014 and 2015.

Variable	Mean 2014	Mean 2015	Z	*p*	Z Corrected	*p*	Number of Series	Number of Tied Series
Ash	12.92	14.24	−1.82522	0.067968	1.771540	0.076472	158	15
Sulfur	0.65	0.66	−2.25469	0.024154	2.201004	0.027736	154	105
Mercury	97.5	78.8	−3.00625	0.002645	2.952567	0.003152	147	69

**Table 2:** Results of the Kolmogorov–Smirnov test of compatibility of distributions in 2014 and 2015.

Variable	Max. Negative Difference	Max. Positive Difference	*p*	Standard Deviation 2014	Standard Deviation 2015
Ash	−0.109195	0.028736	*p* > 0.10	11.22356	12.12545
Sulfur	−0.051724	0.057471	*p* > 0.10	0.28487	0.28874
Mercury	−0.011494	0.224138	*p* < 0.001	54.87257	50.73923

**Table 3:** Results of the Mann–Whitney U test of the compatibility of distributions in 2014 and 2015.

Variable	Sum of Ranks 2014	Sum of Ranks 2015	U	Z	*p*	Z Corrected	*p*
Ash	29,156.50	31,569.50	13,931.50	−1.28522	0.198718	−1.28522	0.198715
Sulfur	30,402.50	30,323.50	15,098.50	0.04156	0.966848	0.04157	0.966842
Mercury	33,561.00	27,165.00	11,940.00	3.40753	0.000656	3.40767	0.000655

**Table 4:** Results of the *t*-tests, F-tests, Levene’s tests, and Brown–Forsythe tests (for 2014 and 2015).

Variable	*t*-test	*p* (Test t)	F-Test	*p* (F-Test)	(L-Test)	*p* (L-Test)	B-F Test	*p* (B-F Test)
Ash	−1.05452	0.292385	1.167171	0.310280	0.520831	0.470975	0.511143	0.475126
Sulfur	−0.15139	0.879756	1.027344	0.859386	0.334133	0.563612	0.180856	0.670903
Mercury	3.30765	0.001040	1.169561	0.303926	0.644871	0.422504	0.759144	0.384201

**Table 5:** Results of the Kruskal–Wallis test of the compatibility of distributions for 2014.

2014	The Test Statistic of the Kruskal–Wallis Test: 451.0681, *p* = 0.000	
	Sum of Ranks	Mean Rank
Ash	46,311.00	266.1552
Sulfur	15,225.00	87.5000
Mercury	74,967.00	430.8448

**Table 6:** Results of the median test of the compatibility of distributions for 2014.

2014	Median Test: Overall Median = 7.36000; Chi-Squared Test Statistic: 348.0000, *p* = 0.000			
	Ash	Sulfur	Mercury	All
≤medians: monitored	87.0000	174.0000	0.0000	261.0000
expected	87.0000	87.0000	87.0000	
monitored-expected	0.0000	87.0000	−87.0000	
>medians: monitored	87.0000	0.0000	174.0000	261.0000
expected	87.0000	87.0000	87.0000	
monitored-expected	0.0000	−87.0000	87.0000	
Total: monitored	174.0000	174.0000	174.0000	522.0000

## Data Availability

The data presented in this study are available online at Supplementary Materials.

## Supplementary Materials

The following are available online at https://www.mdpi.com/article/10.3390/e23070900/s1.
